# Interruption-Aware Computation Offloading in the Industrial Internet of Things

**DOI:** 10.3390/s25092904

**Published:** 2025-05-04

**Authors:** Khoi Anh Bui, Myungsik Yoo

**Affiliations:** 1 Department of Electronic Engineering, Soongsil University, Seoul 06978, Republic of Korea; kbui@soongsil.ac.kr; 2 School of Electronic Engineering, Soongsil University, Seoul 06978, Republic of Korea

**Keywords:** task offloading, edge computing, IIoT, multi-agent deep reinforcement learning

## Abstract

Designing an efficient task offloading system is essential in the Industrial Internet of Things (IIoT). Owing to the limited computational capability of IIoT devices, offloading tasks to edge servers enhances computational efficiency. When an edge server is overloaded, it may experience interruptions, preventing it from serving local devices. Existing studies mainly address interruptions by rerouting, rescheduling, or implementing reactive strategies to mitigate their impact. In this study, we introduce an interruption-aware proactive task offloading framework for IIoT. We develop a load-based interruption model in which the probability of server interruption is formulated as an exponential function of the total computational load, which provides a more realistic estimation of service availability. This framework employs Multi-Agent Advantage Actor–Critic (MAA2C)—a simple yet efficient approach that enables decentralized decision-making while handling large action spaces and maintaining coordination through the centralized critic to make adaptive offloading decisions, taking into account edge availability, resource limitations, device cooperation, and interruptions. Experimental results show that our approach effectively reduces the average total service delay by optimizing the tradeoff between system delay and availability in IIoT networks. Additionally, we investigate the impact of various system parameters on performance under an interruptible edge task offloading scenario, providing valuable insights into how these parameters influence the overall system behavior and efficiency.

## 1. Introduction

The Internet of Things (IoT) connects physical and virtual devices through various communication protocols [1], and its industrial counterpart, the Industrial Internet of Things (IIoT), applies these technologies to industries such as healthcare manufacturing and smart cities [2,3,4,5]. The IIoT integrates industrial automation with advanced computing, machine learning, and communication technologies [6], relying on sensors to collect environmental data for data-driven intelligence. Despite their potential, the resource-limited characteristics of IIoT devices pose significant challenges. Many IIoT devices exhibit restricted computational power, limited battery life, and low storage capacity, rendering local data processing ineffective. Furthermore, the increasing number of IIoT-connected devices escalates data transmission demands, potentially causing network congestion, high latency, and bandwidth limitations [7]. To maintain smooth operation, these conditions require efficient data offloading, caching, and delivery mechanisms. Without system optimization, excessive data burdens can overwhelm cloud servers, slow processing speeds, and extend response times, adversely affecting time-sensitive IIoT applications such as robotic control, smart grid management, and industrial automation.

Task offloading is a computing paradigm that offloads intensive computation tasks from limited resource devices to more powerful external computing platforms, such as a cloud server or edge server closer to the target devices, offering computation speed and real-time responsiveness [8]. Novel solutions that address the strict performance, latency, and reliability requirements of delay-sensitive IIoT applications can be developed by integrating task offloading into IIoT systems [9].

Making optimal offloading decisions requires finding a good tradeoff between minimizing service delay and energy consumption while considering many constrained resources, such as network bandwidth, device capabilities, and server load [10]. Moreover, dynamic changes in the environment with unpredictable network conditions and varying computational loads make proactive task offloading crucial. Reactive approaches often result in higher latency and inefficiencies, whereas proactive strategies anticipate disruptions and optimize resource allocation in advance [11].

Previous research has addressed the issue of task offloading strategies using various methods, ranging from greedy [12] approaches to heuristics [13,14,15,16] and further advanced deep reinforcement learning [17,18,19,20,21] or multi-agent architectures for efficient distributed decision-making [20,22,23]. In addition, the task offloading problem has been integrated with caching [16,17] and resource allocation [14,15,17,24] to make optimal decisions while considering multiple choices and constraint factors. However, the delivery problem has been solved by assuming that the edge server is a high-availability service node. In reality, edge servers with limited resources in real operations may be suspended because of power outages or processing failures when handling too many simultaneous requests. When an interruption occurs, the device’s request must be forwarded to an alternative node source because the current edge node can no longer serve its request. Several studies have discussed the issue of edge interruptions in task offloading [12,18,25]. They solved the problem of interruption-aware task offloading by rescheduling reactive decision-making when an interruption occurred. Although effective, using these methods in real operations may introduce additional delays and reduce the overall system efficiency by performing additional reactive calculations. Moreover, previous studies have used a simple model based on a fixed probability [12] or random probability [26] to simulate the chances of interruption.

To overcome these challenges, this study introduces an interruption-aware, proactive task offloading strategy utilizing a multi-agent deep reinforcement learning-based (MADRL-based) Advantage Actor–Critic (MAA2C) architecture to determine the optimal offloading node for IIoT devices. MAA2C agents continuously learn from their experiences, adapt to the dynamic environment, become aware of the interruption-enabled environment, and adjust their offloading decisions accordingly. We adopt MAA2C as a simple yet efficient and easy-to-implement Actor–Critic framework that supports decentralized execution and naturally handles large discrete action spaces. Compared to value-based multi-agent methods, such as the multi-agent Double Deep Q Network (MAD2QN), which suffer from the problem of action space explosion as the number of choices grows, Actor–Critic methods like MAA2C are more scalable and practical for large-scale multi-agent scenarios. While we fully acknowledge that more advanced techniques, such as multi-agent Proximal Policy Optimization (MAPPO), offer improved stability and performance through centralized critics and clipped policy updates, our goal is to maintain simplicity in the learning process, allowing for a better analysis of the impact of interruptions. By considering edge node availability, network conditions, and workload fluctuations in real time, agents can ensure an optimal tradeoff between service latency and consistent operation of the system. In addition, we propose a realistic load-based interruption model that independently formulates the interruption probability of each edge server based on the total computational load. The contributions of this study are as follows:

- We investigate a case in IIoT edge computing where edge service can be interrupted during operation. We use a load-based exponential function to simulate the interruption probability of edge servers based on their total load. By using this formulation, we can simulate the situation where offloading decisions can lead to interruption, which is more realistic compared to the random-based model.
- We propose a novel task offloading strategy that utilizes a multi-agent deep reinforcement learning-based Advantage Actor–Critic architecture. This approach enables devices to select the optimal computation node by considering factors such as channel status and edge node availability, ensuring an optimal tradeoff between service latency and the consistent operation of the system under interruptions.
- We conduct comparative experiments to assess the effectiveness of the proposed task offloading strategy, and the results demonstrate that our method surpasses its counterparts in terms of total average delay.
- We conduct further experiments to analyze how system parameters impact performance in an interruptible edge task offloading scenario, providing insights into the tradeoffs between service latency, availability, and system stability.

This framework provides a practical solution for computational management in ultra-dense IIoT environments, where edge nodes are vulnerable to interruptions. By employing decentralized decision-making, the system can efficiently handle dynamic workloads and optimize task offloading in real time. Its scalability ensures that it can support a large number of devices, making it well suited for industrial environments with fluctuating resource availability. Additionally, the framework’s consideration of edge node availability and computation capacity provides a robust solution for improving system performance and resource utilization in complex IIoT networks.

The remainder of this paper is organized as follows. In Section 2, we present related studies on task offloading. In Section 3, we introduce the network architecture and discuss some basic system notations for the system models, operation timescale, task offloading decisions, and optimization problem formulation. Section 4 presents the solution to the task offloading problem, where we employ a multi-agent deep reinforcement learning approach based on the Advantage Actor–Critic framework to minimize the total delay. Section 5 describes the implementation of the proposed system and the experimental results. Finally, Section 6 concludes this paper.

## 2. Related Work

Task offloading is essential in edge computing because it distributes computation-intensive tasks from resource-constrained devices to more powerful edge servers. The requirements for task offloading are typically formulated as optimization problems with multiple objectives and constraints. To minimize the service latency, a previous study solved the problem of task offloading based on user association decisions [13]. First, they used a heuristic approach to obtain the optimal user association decision; then, the tasks were partitioned to assign each user as a mixed-integer programming problem. In [14], the authors explored a digital twin-empowered edge computing architecture in which multiple user equipment systems offload computing tasks to edge servers based on an edge-selection decision. They proposed an iterative optimization algorithm that relaxed integer constraints and optimized the power allocation, edge selection, offloading policies, and processing rates using convex programming techniques. In [16], a joint strategy was introduced for user association, service caching, and task offloading to reduce delays and improve the quality of service in multitier communication and edge computing heterogeneous networks. They used a joint delay-aware approach to solve the problems of user association, service caching, and task offloading by choosing a base station with the maximum signal-to-noise ratio and minimum total delay. These studies optimize task offloading through user association, edge node selection, and service caching, but they overlook the resource allocation. Resource allocation plays a critical role in jointly optimizing offloading decisions and system performance.

Other studies have focused on overcoming network delay challenges and optimizing resource allocation in cloud–edge collaborations. One such study [17] overcame the challenge of network delay in cloud–edge collaboration networks by solving the joint problem of caching, delivery, and computational resource allocation using a Q-learning-based delay-aware approach. In another study [15], a joint task offloading and resource allocation scheme was proposed for accuracy-aware machine learning-based IIoT applications in an edge–cloud network. Their goal was to minimize the long-term average system cost while considering task offloading, computing resource allocation, and machine learning inference accuracy. They used Lyapunov optimization and Benders decomposition to achieve an optimal solution, and a heuristic algorithm for efficient computation was applied to solve this problem. In [19], the authors studied online task offloading with diverse users in mobile edge computing networks to minimize overall task latency. Unlike traditional methods that assume stable task arrivals and homogeneous users, this method considered time-variant task generation and varying security and performance requirements. A novel collaborative architecture was proposed by classifying edge nodes into public and private categories and developing a matrix geometric solution framework with a deep reinforcement learning-based online task scheduling algorithm. These studies effectively addressed the joint optimization of task offloading and resource allocation to improve system performance. However, they mainly focused on single-hop offloading and did not consider collaborative processing across multiple nodes or multi-hop task offloading strategies.

Several studies have explored multi-hop and collaborative computing scenarios. In [24], a multistage Stackelberg game model was proposed for optimizing task offloading and resource allocation in vehicular edge computing by incentivizing service vehicles to share computing resources through lease contracts. Adaptive lease contracts and multiarmed bandit-based selection algorithms help service vehicles determine optimal strategies. Simultaneously, roadside units act as buyers and sellers of computational resources. Ref. [20] described a framework for hierarchical multi-hop task offloading and relay selection in IoT edge computing. The devices in coverage are divided into layers based on the distance from the central edge server, and they then use a Soft Advantage Actor–Critic reinforcement learning agent to sequentially choose the next relay to offload the task in the direction toward the central edge. The results are then sent back to the device via a routing path. The research in [21] investigated task offloading in UAV-aided wireless-powered edge computing to enhance the freshness of information, while ensuring UAV energy safety. A constrained Markov decision process was formulated to jointly optimize the wireless charging power, UAV trajectory, and offloading decisions with a safe deep Q-network algorithm enforcing energy constraints via Lyapunov equations.

Existing studies have solved the problem of task offloading in different ways, extending from greedy approaches to heuristic and state-of-the-art deep reinforcement learning (DRL) approaches. Among these, DRL-based approaches have demonstrated significant advantages owing to their ability to handle dynamic environments, optimize sequential decision-making, and adapt to changing network conditions [27]. DRL systems can proactively improve the offloading efficiency by learning optimal policies from experience rather than relying on fixed rules. They can also combine the task offloading problem with caching or resource allocation problems to achieve optimal joint decisions while ensuring many constraint factors. However, in ultra-dense networks, decentralized and distributed dynamic decision-making must be considered to improve scalability and overall system performance. Multi-agent DRL-based approaches are a promising solution.

In [23], the authors propose a decentralized two-stage multi-agent DRL framework for task offloading and resource management in wireless powered MEC networks, where high-level agents at access points optimize energy transmission, and low-level agents at devices independently decide offloading strategies and local computing parameters to adapt to dynamic environments. Multi-agent architectures, such as MAA2C, are leveraged in [22] to enable decentralized task offloading in Industrial IoT environments. By establishing a Markov decision model with a constrained action space and adopting a Centralized Training Decentralized Execution framework, the proposed approach reduces system delay and adapts effectively to heterogeneous and dynamic industrial settings. MAPPO (a more stable advancement of MAA2C) was used in [28] to address the challenges of real-time decision-making in IIoT. The authors proposed a two-stage method called MCORM, where resource allocation is solved using a combinatorial bandit algorithm, and task offloading decisions are made through distributed multi-agent MAPPO, enabling efficient and scalable task management across devices.

The above studies only considered cases in which the edge node or service provider node could operate smoothly without any suspension. In a real operational scenario, many factors can cause edge servers to fall because they cannot serve device requests owing to power, system, or computational bottlenecks. Several studies have investigated the impacts of edge server disruptions. In [12], the authors investigated task scheduling in task offloading scenarios, where computational tasks were modeled as directed acyclic graphs. They aimed to minimize task response delay while ensuring failure resilience. Assuming a fixed interruption probability for edge servers, they proposed a failure-resilient scheduling approach, including a context-aware greedy task scheduling algorithm and a dependency-aware task rescheduling mechanism. The experimental results demonstrated that their approach effectively reduced task completion time and mitigated scheduling interruptions caused by server failures. In [26], the tradeoff between bandwidth consumption and reliability in IoT applications was investigated, where tasks were offloaded onto containers or virtual machines with certain failure and recovery rates. They modeled these rates as a random Poisson process and formulated a multiobjective optimization problem to minimize the bandwidth usage while maximizing the reliability. Their work introduced two efficient approaches to transform the problem into a single-objective optimization, achieving near-optimal solutions. In [18], the advantages of parking vehicles with idle resources were used to offload latency-sensitive tasks to the Internet of Vehicles. They focused on the problem of communication links being interrupted owing to frequent changes in vehicle availability, which raises the challenge of heterogeneity in resources and parking vehicle network topologies.

Existing studies have primarily relied on random- or fixed-value simulations to model edge server interruptions. However, these models lack realism because the likelihood of an interruption is inherently tied to the workload and resource utilization of the server. Moreover, current approaches primarily focus on reactive strategies, such as rescheduling tasks after an interruption occurs. Although this approach can mitigate the immediate effects of interruptions, it suffers from increased delays and inefficiencies owing to the need for real-time adjustments and reallocation. DRL-based approaches offer a promising solution because they can learn from experience and make proactive, predictive decisions to prevent overload-induced failures, thereby reducing the need for frequent rescheduling and improving system stability. This paper presents an interruption-aware task offloading approach that can make adaptive offloading decisions by considering edge availability, resource limitations, device cooperation, and awareness of interruptions. Additionally, we present a load-based interruption model that expresses the likelihood of a server interruption as an exponential function of the overall computational load, leading to a more accurate simulation of service availability. A summary of the contributions of recent studies is presented in Table 1.

## 3. System Model

**Figure 1:**
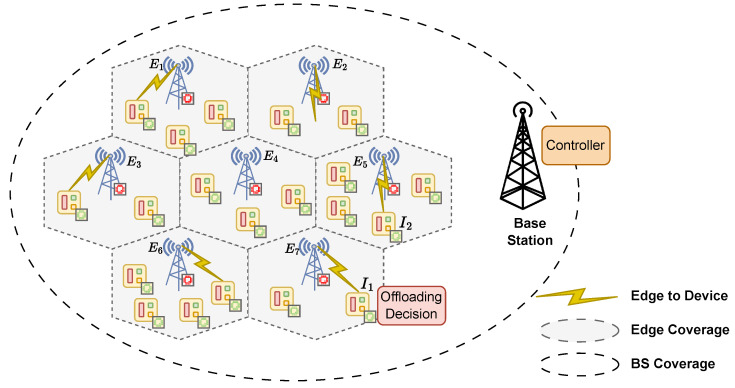
Network architecture.

The IIoT network considered in this study consists of *N* edge servers $\left\{E_{1},E_{2},\ldots ,E_{N}\right\}$ deployed in a hexagonal coverage grid formation with IIoT devices randomly distributed in their coverage. All the devices and edge servers operate under the coverage of an IIoT base station (BS) that functions as a network controller, as shown in Figure 1. The set of IIoT devices is denoted as $\left\{I_{1},I_{2},\ldots ,I_{M}\right\}$, where *M* denotes the number of devices. The system operates in a *T* time slot model, where each time slot t∈T represents a decision-making slot. At each time slot *t*, IIoT devices generate computation tasks that must be processed. To minimize computational latency, tasks are offloaded to the edge servers, which are assumed to have more powerful computational capabilities for efficient execution while preventing excessive delays.

In this framework, each IIoT device acts as an independent agent, making task offloading decisions based on real-time system-state information from the BS. The BS collects the channel status and edge server availability and shares relevant data with the devices. After a device selects where to process its task, the BS communicates with the chosen edge server to pre-allocate the computational resources. The devices then send their task data to the selected edge server for computational offloading or local handling. In real systems, the BS controller can quickly notice if a server has a problem by using simple methods such as heartbeat signals or periodic status updates [29]. Therefore, we assume that, if an interruption occurs at any edge server during operation, the BS server controller can promptly detect it. The BS then updates the task routing if a relay node is interrupted along a wired data transmission path. Alternatively, if the offloaded node is interrupted, the BS triggers on-device processing by signaling the device.

### 3.1. Task Offloading and Computation Delay Model

In an IIoT environment, devices continuously generate computational tasks that require processing within a limited period. At each time slot *t*, device $I_{i}$ generates a computational task, defined as follows:

(1) $$\tau_{i}\left(t\right)=\left\{s_{i}\left(t\right),f_{i}\left(t\right),r_{i}\left(t\right)\right\},$$

where $s_{i}\left(t\right)$ represents the task size in bits, $f_{i}\left(t\right)$ denotes the number of CPU cycles required to complete each bit of the task, and $r_{i}\left(t\right)$ is the deadline for task completion. The total required load of device $I_{i}$ at time slot *t* is defined as

$$C_{i}\left(t\right)=s_{i}\left(t\right)\times f_{i}\left(t\right).$$

Then, each device $I_{i}$ can decide where to execute its task: locally on the device, on its local edge server, or on a remote edge server. We define $local_{i}$ as the index that indicates the local edge server of device $I_{i}$. The offloading decision is represented by a binary variable $X_{i,j}\left(t\right)\in \left\{0,1\right\}$, defined as follows:

(2) $$X_{i,j}\left(t\right)=\left\{\begin{array}{cc} 1, & \mathrm{if}\;\mathrm{task}\;\tau_{i}\;\mathrm{is}\;\mathrm{executed}\;\mathrm{at}\;j,\\ 0, & \mathrm{if}\;\mathrm{task}\;\tau_{i}\;\mathrm{is}\;\mathrm{not}\;\mathrm{executed}\;\mathrm{at}\;j. \end{array}\right.$$

The index *j* can assume values in {0,1,2,…,N}. With $X_{i,j}\left(t\right)=1$, if j=0, the task is processed locally on the device instead of being offloaded. If j∈[1,N], the task is executed on the edge server $E_{j}$ (when $j=local_{i}$, task $\tau_{i}$ is offloaded to the local edge server of its device).

Each IIoT device has a computation capacity of $F_{D}$ cycles per second, and the edge servers are assumed to have more powerful computation processors with a computation capacity of $F_{E}$ ($F_{E}>F_{D}$). The computation delay for device $I_{i}$ when computing the task on the device can be calculated as

(3) $$D_{i}^{comp,0}\left(t\right)=\frac{C_{i}\left(t\right)}{F_{D}}.$$

Edge servers allocate computational capacity equally among all device-offloading tasks with a maximum capacity limit of $F_{E}^{max}$. The use of a straightforward equal allocation strategy, rather than dynamic resource management, is intended to keep the model simple and focus on demonstrating the effect of server interruptions on offloading decisions. While dynamic allocation could improve processing efficiency, it would also make the model much more complicated. We consider adding a more advanced resource allocation method as an extension for future work.

The number of devices that offload their tasks to the edge server $E_{j}$ at time slot *t* is given by

(4) $$N_{j}\left(t\right)=\sum_{i}X_{i,j}\left(t\right).$$

Then, the computation delay for device $I_{i}$ when offloading its task to edge server $E_{j}$ at *t* can be defined as

(5) $$D_{i}^{comp,j}\left(t\right)=\frac{C_{i}\left(t\right)}{\min(F_{E}^{max},F_{E}/N_{j}\left(t\right))}.$$

The total computation load ratio of the edge server $E_{j}$ at time *t* can be computed as follows:

(6) $$L_{j}\left(t\right)=\frac{\sum_{i=1}^{M}C_{i}\left(t\right)*X_{i,j}\left(t\right)}{F_{E}}.$$

### 3.2. Transmission Delay Model

In this scenario, the IIoT devices can connect to their local edge servers through direct wireless cellular links. Each edge server provides devices within its coverage area with a dedicated link with a fixed spectrum of $B_{E}$ MHz using the orthogonal frequency-division multiplexing technique, contributing to a total bandwidth of $B_{E}^{total}$ MHz. Because edge server coverage does not overlap and adjacent cellular coverage operates on distinct spectrum ranges, there is no inter-cell interference. The uplink data rate between device $I_{i}$ and edge server $E_{j}$ at time slot *t* is expressed as the Shannon channel capacity, as shown in [30,31]:

(7) $$R_{i,j}\left(t\right)=B_{E}\log_{2}\left[1+\frac{P_{E}*PL_{i,j}\left(t\right)}{\sigma^{2}}\right],$$

where $P_{E}$ is the edge server transmission power and $PL_{i,j}$ is the path loss between device $I_{i}$ and edge server $E_{j}$. To represent the wireless channel status of IIoT devices, let $H\in R^{M}$ be the channel status matrix, where H[i] is the channel gain from the local edge server. In our work, devices are randomly distributed within the local edge coverage, and no mobility is considered; therefore, the main varying factor between devices is the distance from their local edge. Therefore, by considering only the path loss model due to distance, which is a major factor compared to small-scale fading, we can simplify the channel estimation, as it still captures the dynamics of the channel status. Incorporating small-scale fading effects is a crucial extension that could be considered in future work to further enhance the accuracy of throughput estimation. Only the uplink data rates are formulated, whereas the downlink delay is not considered because the task results are very small compared to the task size and can be retrieved almost instantly from the remote edge [32,33,34,35,36,37]. The communication between the two edge servers is a wired optic fiber connection with a fixed-size data rate $R_{E2E}$.

The wireless transmission delay for device $I_{i}$ to offload its task to its local edge server can be computed as follows:

(8) $$D_{i}^{wireless}\left(t\right)=\frac{s_{i}\left(t\right)}{R_{i,j}\left(t\right)}.$$

When the device forwards the task to a remote edge server $E_{j}$, an additional transmission delay occurs owing to the data transfer between the edge servers over the wired connection. The delay when transmitting task data $s_{i}\left(t\right)$ between the remote edge servers $E_{u}$ and $E_{v}$ can be computed as

(9) $$D_{u,v}^{wired}\left(t\right)=\frac{s_{i}\left(t\right)}{R_{E2E}}\times h_{u,v},$$

where $h_{u,v}$ represents the shortest hop distance in the wired network path between two edge servers $E_{u}$ and $E_{v}$.

### 3.3. Load-Based Interruption Model and Interruption Delay

#### 3.3.1. Interruption Probability

**Figure 2:**
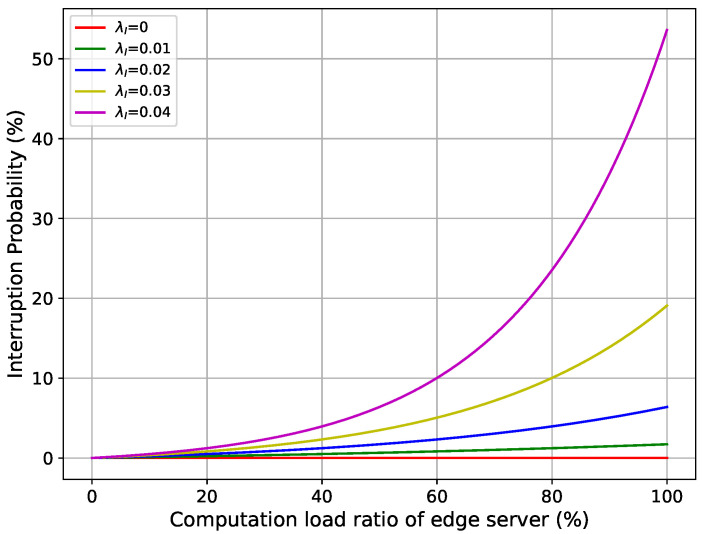
Interruption probability $P_{j}^{int}\left(t\right)$ for different interruption sensitivity values ($\lambda_{I}$).

In the real world, edge servers that handle excessive user requests may suffer from network congestion, increased latency, or temporary service disruptions due to computational resource exhaustion [38,39,40,41]. Instead of formulating the interruption probability with a random distribution and treating them as peripheral factors, as in [26], in this study, we investigate a case in which task offloading decisions directly influence the likelihood of edge server interruptions. To achieve this, we adopt a simplified failure model in which the probability of an edge server experiencing an interruption is based on its total computational load. The interruption probability for each edge server $E_{j}$ can be formulated as an exponential function, as follows:

(10) $$P_{j}^{int}\left(t\right)=e^{\lambda_{I}\times L_{j}\left(t\right)}-1,$$

where $L_{j}\left(t\right)$ represents the load ratio of the edge server $E_{j}$, which can be computed using Equation (6), and $\lambda_{I}$ is the sensitivity parameter of the exploration function. The interruption probabilities for different values of $\lambda_{I}$ are illustrated in Figure 2. Even with a very small load ratio, the edge servers suffer from a low chance of interruption (see Figure 2). In a computing system, in addition to hardware and software issues, which are the primary causes of node interruptions, there are also minor adverse factors such as human errors, environmental influences, network problems, and even unpredictable unknown events [42]. Compared to linear or logistic models, the exponential function provides a more realistic estimation of edge server interruptions. A linear model assumes that the chance of interruption increases steadily as the load grows; however, this does not accurately reflect reality, as systems typically remain stable when the load is low or moderate, and only become unstable when the load is extremely high. A logistic model exhibits a slowdown or saturation effect, which is suitable for systems that have mechanisms to control instability. However, in edge computing, where resources are limited, interruptions often increase very quickly without slowing down. The exponential model better captures this sudden rise; even a small increase in load at high usage can cause the system to fail much faster. The sensitivity parameter $\lambda_{I}$ allows for the flexible adjustment of the interruption probability’s growth speed, as shown in Figure 2.

Let $Z\left(t\right)\in R^{N}$ be a binary matrix representing the availability of status edge servers, where

(11) $$Z_{j}\left(t\right)=\left\{\begin{array}{cc} 0, & \;\mathrm{if}\;\mathrm{edge}\;\mathrm{server}\;E_{j}\;\mathrm{is}\;\mathrm{available},\\ 1, & \;\mathrm{if}\;\mathrm{edge}\;\mathrm{server}\;E_{j}\;\mathrm{is}\;\mathrm{interrupted}. \end{array}\right.$$

Owing to variations in transmission and computation delays, offloaded tasks at the edge server start and finish at different times within a given period. Consequently, the server load fluctuates over time, dynamically affecting the interruption probability $P_{j}^{int}\left(t\right)$. A random sample $U_{j}\left(t\right)∼U\left(0,1\right)$ is independently drawn for each edge server $E_{j}$ at the beginning of each time slot to sample the interruption event. Whenever a server’s load changes within the time slot $P_{j}^{int}\left(t\right)$ using Equation (10), and the interruption state is redetermined using the pre-sampled $U_{j}$,

(12) $$Z_{j}\left(t\right)=\left\{\begin{array}{cc} 0, & \;\mathrm{if}\;U_{j}\left(t\right)\geq P_{j}^{int}\left(t\right),\\ 1, & \;\mathrm{if}\;U_{j}\left(t\right)<P_{j}^{int}\left(t\right). \end{array}\right.$$

If an interruption occurs at any edge server, all unfinished tasks at this edge node will be recalculated by their corresponding local devices with the same extra interruption delay as this node, which will be described in the following Section 3.3.2. Interrupted edge servers will then become unavailable for the next $\Delta_{int}$ time slots.

#### 3.3.2. Interruption Delay Model

At each time slot *t*, tasks are generated and transmitted simultaneously. At the edge server $E_{j}$, owing to the different transmission paths and channel statuses, they arrive at different times, gradually increasing the server load $L_{j}\left(t\right)$. $P_{j}^{int}\left(t\right)$ also increases with the load. When the interruption probability $P_{j}^{int}\left(t\right)$ surpasses the random variable $U_{j}\left(t\right)$, the server is interrupted, forcing all ongoing computations to return to their local device hardware. This fallback incurs ongoing devices with the same additional delay, equal to the arrival time of a specific task $\tau_{i^{*}}$, which leads to a change in load, ultimately triggering an interruption:

- If the triggering task comes from a device within the coverage area of edge server $E_{j}$ ($local_{i^{*}}=j$), the interruption delay is
  (13) $$D_{j}^{int}\left(t\right)=D_{i^{*}}^{wireless}\left(t\right)$$
- If the task was offloaded from a remote device ($local_{i^{*}}\neq j$), the interruption delay includes both wireless and wired transmission delays:
  (14) $$D_{j}^{int}\left(t\right)=D_{i^{*}}^{wireless}\left(t\right)+D_{local_{i^{*}},j}^{wired}\left(t\right),$$
  where $i^{*}$ represents the index of the device whose task arrival updated the server’s load and triggered the interruption, and $local_{i^{*}}$ is the index of the local edge server of device $I_{i^{*}}$.

**Figure 3:**
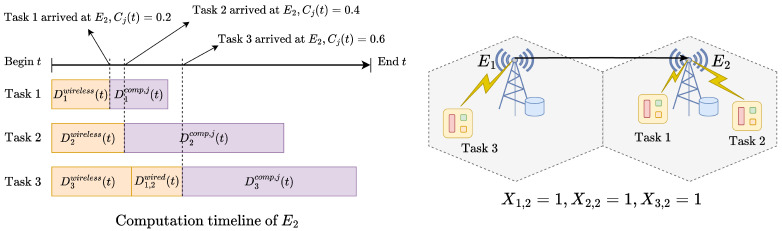
Edge server computation timeline for a simple scenario with two edge servers and three IIoT devices with $X_{1,2}=1,X_{2,2}=1,$ and $X_{3,2}=1$.

For example, in the simple scenario shown in Figure 3, the interruption can occur at any time the server computation load is accumulated by task arrivals:

- If $E_{2}$ is interrupted when *Task 2* arrives, the interruption delay $D_{2}^{int}\left(t\right)$ can be computed using Equation (13) with $i^{*}=2$ and *Task 1*, and *Task 2* must be recomputed at local devices with the same additional delay $D_{2}^{int}\left(t\right)$.
- If $E_{2}$ is interrupted when *Task 3* arrives (from $E_{1}$), the interruption delay $D_{2}^{int}\left(t\right)$ is computed using Equation (14) with $i^{*}=3$. *Task 2* and *Task 3* must fallback to the local device computation. However, in this case, *Task 1* is already finished before the interruption, so it is not affected by the interruption.

### 3.4. Total Delay Model

#### 3.4.1. Total Delay Model with No Interruption

The total delay of device $I_{i}$ when offloading its task at *t* is denoted by $D_{i}^{total}\left(t\right)$, which is affected by the task offloading decision $X_{i,j}\left(t\right)$. When no interruption occurs, the total delay for different cases can be formulated as follows:

- When $X_{i,0}\left(t\right)=1$, device $I_{i}$ processes the task using its hardware. In this case, the total delay can be computed as the processing time on the local device:
  (15) $$D_{i}^{total}\left(t\right)=D_{i}^{comp,0}\left(t\right).$$
- When $X_{i,local_{i}}\left(t\right)=1$, device $I_{i}$ offloads the task to its local edge server. In this case, the total delay can be computed as the sum of the wireless transmission delay and the computation delay at the local edge:
  (16) $$D_{i}^{total}\left(t\right)=D_{i}^{wireless}\left(t\right)+D_{i}^{comp,local_{i}}\left(t\right)$$
- When $X_{i,j}\left(t\right)=1$, and $j\in \left\{1,2,\ldots ,N\right\}\setminus local_{i}$, device $I_{i}$ offloads the task to remote edge server $E_{j}$. In this case, the device experiences wireless transmission delay, multi-hop wired transmission delay, and remote edge computation delay.
  (17) $$D_{i}^{total}\left(t\right)=D_{i}^{wireless}\left(t\right)+D_{local_{i},j}^{wired}\left(t\right)+D_{i}^{comp,j}\left(t\right).$$

#### 3.4.2. Total Delay Model with Computation Node Interruption

It is important to note that when a server experiences an interruption during task processing, it introduces additional delays for all tasks that remain unfinished. In such cases, the tasks must be processed by the local device, which suffers from high computational latency due to its poorer capabilities compared to edge nodes. The chance of server interruption increases dynamically as the server load rises (Equation (10)), meaning that task latencies are highly sensitive to the system’s load status.

When the offloaded edge server $E_{j}$ is interrupted, all unfinished tasks return to the local device, incurring an extra delay. The total delay in this case is given by

(18) $$D_{i}^{total}\left(t\right)=D_{j}^{int}\left(t\right)+D_{i}^{comp,0}\left(t\right),$$

where $D_{j}^{int}\left(t\right)$ represents the interruption delay of the affected edge server $E_{j}$, which can be calculated using either Equation (13) or (14), depending on the device that caused the interruption, and $D_{i}^{comp,0}\left(t\right)$ is the computation time required for on-device processing after the fallback.

#### 3.4.3. Total Delay Model with Relay Node Interruption

In addition to disrupting the computation at the edge node, interruptions affect the multi-hop wired transmission path. Local edge nodes act as relay points, enabling communication between their covered devices and remote edge servers. The availability and reliability of these relay nodes within the multi-hop transmission path play a critical role in shaping the network topology and, consequently, the transmission delay. If device tasks cannot reach the intended remote nodes due to disruptions in the relay network, these tasks must be processed locally on the device, resulting in an additional delay.

**Figure 4:**
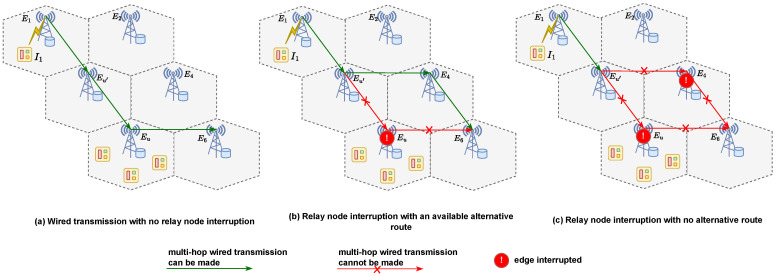
Task data-wired transmission during remote offloading under different scenarios (with $X_{1,6}=1$). (**a**) Wired transmission with no relay node interruption, (**b**) relay node interruption with an available alternative route, and (**c**) relay node interruption with no alternative route.

If any relay edge server $E_{u}$ along the wired data transmission path is interrupted, the BS server must either reroute the data through an alternative route or return it to the local computation. If an alternative route is available from the preceding node $E_{u^{\prime }}$ before the interruption to the target edge $E_{j}$ (see Figure 4b), the total delay is given by

(19) $$D_{i}^{total}\left(t\right)=D_{u}^{int}\left(t\right)+D_{u^{\prime },j}^{wired}\left(t\right)+D_{i}^{comp,j}\left(t\right),$$

where $D_{u}^{int}\left(t\right)$ is the interruption delay of the affected relay server $E_{u}$, and $D_{u^{\prime },j}^{wired}\left(t\right)$ represents the wired transmission delay from the last successful relay node $E_{u^{\prime }}$ to the target computation server $E_{j}$. However, if no alternative path is available (Figure 4c), the computation must return to the local device, resulting in an extra delay:

(20) $$D_{i}^{total}\left(t\right)=D_{u}^{int}\left(t\right)+D_{i}^{comp,0}\left(t\right).$$

These interruption-induced delays significantly affect the task execution time, highlighting the need for intelligent task allocation strategies that consider network conditions and potential interruptions.

### 3.5. Problem Formulation

Multiple factors, including channel conditions and edge node status, influence the task offloading efficiency in IIoT edge caching. In this section, the task offloading decision problem is formulated as an optimization problem with the aim of minimizing the average total delay $D_{i}^{total}\left(t\right)$ while satisfying the following constraints:

(21) $$\begin{array}{cc} \min_{X_{i,j}\left(t\right)}\; & \frac{1}{T}\frac{1}{M}\sum_{t=1}^{T}\sum_{i=1}^{M}D_{i}^{total}\left(t\right) \end{array}$$

(22) $$\begin{array}{cc} s.t.\; & \left(C1\right):\sum_{i}R_{i,j}\left(t\right)\leq B_{E}^{total},\;\forall j\in \left\{1,2,\ldots ,N\right\} \end{array}$$

(23) $$\begin{array}{cc} \; & \left(C2\right):\sum_{i}X_{i,j}\left(t\right)C_{i}\left(t\right)\leq F_{E},\;\forall j \end{array}$$

(24) $$\begin{array}{cc} \; & \left(C3\right):X_{i,j}\left(t\right)\leq 1-Z_{j}\left(t\right),\;\forall i,j\; \end{array}$$

(25) $$\begin{array}{cc} \; & \left(C4\right):X_{i,j}\left(t\right)\in \left\{0,1\right\},\;\forall i,j\; \end{array}$$

(26) $$\begin{array}{cc} \; & \left(C5\right):D_{i}^{total}\left(t\right)\leq \tau_{i}\left(t\right),\;\forall i,t \end{array}$$

(27) $$\begin{array}{cc} \; & \left(C6\right):\sum_{j=0}^{N}X_{i,j}\left(t\right)=1,\;\forall i,t. \end{array}$$

The total transmission delay $D_{i}^{total}\left(t\right)$ in the objective function (21) can be computed in different ways (Equations (15)–(20)), depending on the delivery decision $X_{i,j}\left(t\right)$ and the interruption state. Constraint (22) ensures that the total bandwidth allocated to the devices offloading to a given edge server does not exceed the maximum available bandwidth. Constraint (23) guarantees that the total computational load assigned to each edge server does not exceed the available computational capacity. Constraint (24) indicates that a task can only be offloaded to an available edge server (i.e., it is not interrupted). Constraint (25) ensures that the offloading decision variable is binary, that is, the task is either offloaded to a specific edge server or not. Constraint (26) ensures that each task is completed within the required deadline. Finally, Constraint (27) guarantees that each device can offload its task to at most one edge server at any time *t*.

## 4. Multi-Agent Deep Reinforcement Learning for Task Offloading

Traditional approaches to this problem suffer from critical limitations, including a lack of system-state awareness and the inability to learn from experience. Although reinforcement learning offers a promising solution, single-agent methods struggle with the curse of dimensionality and the challenges of distributed decision-making. To address these issues, we adopted a Multi-Agent Advantage Actor–Critic framework [43] to optimize the task offloading decision $X_{i,j}\left(t\right)$. This approach mitigates action space complexity while enabling cooperative decision-making among agents. By leveraging centralized training with decentralized execution (CTDE) and a shared critic network, the proposed method ensures scalable, adaptive, and efficient learning in a dynamic IIoT environment.

Unlike traditional heuristics, MAA2C agents learn proactively from experience patterns such as overload and congestion during training, refining policies to balance total delay and stability, and making adaptive decisions in an interruption-enabled environment. Because the agents are homogeneous, utilizing a shared-parameter critic network facilitates experience sharing and cooperative decision-making among the agents [44,45,46,47]. This adaptive decision-making improves system performance and enhances overall network efficiency.

### 4.1. Partially Observable Markov Decision Process

When using MAA2C, the task offloading problem in a dynamic IIoT environment can be formulated as a decentralized partially observable Markov decision process (Dec-POMDP), represented as a tuple:

(28) M=〈I,∫,a,P,∇,γ〉,

where I={1,2,…,M} represents the set of agents corresponding to IIoT devices; $\int =\{o_{1},o_{2},\ldots ,o_{M}\}$ is the global state space of the environment, which is not fully observable by individual agents; $a=\{a_{1},a_{2},\ldots ,a_{M}\}$ is the joint action set; and γ∈(0,1] is the discount factor that determines the importance of future rewards.

#### 4.1.1. Observation Space

Because each agent *j* in the Dec-POMDP setting has only partial knowledge of the environment, it maintains a local observation $o_{i}\left(t\right)\in \int \left(t\right)$ at each time step *t*, defined as

(29) $$o_{i}\left(t\right)=\left\{\tau_{i}\left(t\right),Z\left(t\right),H_{i}\left(t\right),V_{i}\left(t\right)\right\},$$

where $\tau_{i}$ is the task information, Z(t) denotes the interruption state of the system, $H_{i}\left(t\right)$ denotes the wireless channel condition between device $I_{i}$ and its local edge server, and $V_{i}$ represents the shortest hop distance of device $I_{i}$ to every other edge server.

#### 4.1.2. Action Space

At each time step *t*, agent *i* selects an action $a_{i}\left(t\right)$ from the discrete action set:

(30) $$a_{i}\left(t\right)\in \left\{0,1,2,\ldots ,N\right\},$$

which corresponds to the task offloading decision. Specifically, $a_{i}\left(t\right)=0$ means device *i* processes its task on its own local hardware, and $a_{i}\left(t\right)=j\in \left\{1,2,\ldots ,N\right\}$ denotes that the task is offloaded to server *j*. Notably, when $j=local_{i}$, device *i* offloads its task to the local edge server.

#### 4.1.3. State Transition Probability

The probability P(∫(t+1)∣∫(t),a(t)) represents the probability of transitioning from the current state ∫(t) to a subsequent state ∫(t+1) after performing joint action a(t).

#### 4.1.4. Reward Function

The reward function consists of two key components that guide the agents toward optimal decision-making while ensuring cooperation between agents. This involves a shared reward term that minimizes the average transmission delay, which can be obtained after all delivery and routing decisions, and a penalty term that discourages actions leading to network interruptions.

The reward function is given by

(31) $$\nabla_{i}\left(t\right)=-\alpha \left[\frac{1}{M}\sum_{i}D_{i}^{total}\left(t\right)\right]-\beta \phi_{1}-\phi_{2},$$

where α is a scaling factor for the shared reward and β is a scaling factor for the penalty term $\phi_{1}$, which is an extra penalty applied when the selected decision leads to an interruption. Constraint (26) is enforced by the penalty term $\phi_{2}$. This reward formulation encourages cooperation among agents by minimizing the average transmission delay while penalizing actions that disrupt network operations.

### 4.2. Agent Training and Execution

In the CTDE setting, agents are trained in a centralized manner but execute decisions in a decentralized fashion. This approach allows the system to take advantage of shared knowledge using a shared-parameter critic network during training, while enabling individual agents to act independently during execution. The training process consists of updating both the actor and critic networks to optimize the policy for each agent. During training, the shared critic network observes the joint state ∫ of the system and evaluates the state-action value Q(∫(t),a(t)) for all the agents, allowing for a more informed and stable value estimation. However, during execution, only the actor networks are required for decision-making. Each agent selects its action based on its local observation $o_{i}\left(t\right)$ without requiring access to the global state or the information of other agents [43]. This decentralized decision-making process ensures that each agent operates autonomously while benefiting from a collaborative training phase. Additionally, invalid actions are masked during operation to ensure that the agents choose only feasible decisions.

## 5. Experiment

### 5.1. Experimental Setup

In this section, an evaluation of the performance of the proposed method using a quantitative simulation is presented. The detailed default hyperparameter setups for the simulation and MAA2C agent training are described in Table 2. All experiments were conducted on a Linux server equipped with an Intel Core i7-10700K processor, 32 GB of RAM, and an RTX 2060 GPU with 6 GB of VRAM. The implementation was developed in Python 3.10.14, and the MAA2C model was built using the PyTorch 2.6 deep learning framework.

#### 5.1.1. Comparison Counterpart Setup

The candidates used in the comparison experiments are described as follows:

- ***MAA2C-based*** **(#1—ours)**: This is our proposed task offloading strategy that uses a MAA2C-based approach to solve the offloading optimization problem to minimize the total delay with the proposed load-based interruption model.
- ***MAD2QN*** **(#2)**: A multi-agent version of the Double Deep Q-Network (D2QN), where each agent independently learns a Q-function while using double Q-learning techniques to stabilize training. This approach addresses the same optimization problem as **#1** but uses value-based learning instead of Actor–Critic methods.
- ***A2C*** **(#3)**: A single-agent Advantage Actor–Critic (A2C) method where a centralized controller is responsible for offloading decisions for all devices. This baseline highlights the difference between centralized and decentralized decision-making in multi-agent environments.
- ***Greedy*** **(#4)**: A heuristic-based offloading strategy where tasks are prioritized based on their required computational cycles per second ($C_{i}/r_{i}$). Devices first attempt to offload to the least-loaded, nearby available servers. If no servers are suitable, tasks are processed locally.
- ***Random*** **(#5)**: Within this approach, offloading decisions are randomly made.
- ***LocalEdge*** **(#6)**: This strategy restricts the IIoT device to offload its task to the local edge server.
- ***OnDevice*** **(#7)**: The IIoT device only uses its hardware to compute the tasks.

The simulation was conducted in an industrial zone with servers deployed in a hexagonal coverage formation, covering an area of 500 m. The comparison experiment was conducted with the following default parameters: N=5 edge servers, M=20 devices, $\lambda_{I}=10^{-2}$, and $\Delta_{int}=5$ s, and run for T=3000 time slots. It is important to note that there is no optimal value for $\lambda_{I}$. Based on Figure 2, as the $\lambda_{I}$ value increases, the environment becomes more sensitive to interruptions. This can be observed as an inverse relationship between average total delay and $\lambda_{I}$, where higher $\lambda_{I}$ values lead to increased interruptions and, consequently, longer delays. Therefore, we use a fixed general value of $\lambda_{I}$ for other parameter analyses.

#### 5.1.2. Evaluation Metrics

The performance evaluation indicators selected for our simulation are as follows:

- Average total delay (*Avg. Delay*) in milliseconds. This is the value of the objective function, which can be computed using Equation (21).
- Availability score (*Avail.*) in percentage, which monitors the availability status of an edge server:
  (32) $$Availability=\frac{1}{T}\sum_{t\in T}\sum_{j\in N}\frac{Z_{j}\left(t\right)}{N}\times 100.$$

### 5.2. Experiment Results and Analysis

#### 5.2.1. Impact of Interruption on Simulation Results

To evaluate the impact of interruptions on system performance, we conducted a comparative experiment to compare the proposed *MAA2C-based* model with various methods in both normal and interrupted scenarios. In this experiment, the hyperparameters were set to their default values. The experimental results are listed in Table 3.

With $\lambda_{I}=0$ (no interruption), the problem becomes a straightforward task of offloading decision-making. The *LocalEdge* method achieved the best performance with an *Avg. Delay* of 272.70 ms because the edge servers remain available, reducing wired transmission delays by eliminating the need to offload tasks to remote edge servers. The *MAA2C-based* method achieved the second-best performance with an *Avg. Delay* of 273.43 ms. Compared to more complex processes, such as DRL-based or *Greedy*, simply offloading tasks to the local edge server appears to be the best solution in these cases.

However, when interruptions are introduced into the system, the *MAA2C-based* method outperformed all other methods with an *Avg. Delay* of 390.95 ms and an *Availability* of 83.07%. The *MAA2C-based* method can efficiently distribute tasks among edge servers while balancing local computation and offloading. Due to the interruption, system performance was significantly affected, increasing *Avg. Delay* from 273.43 to 390.95 ms. This increase occurred because, upon interruption, the devices must restart the computation locally from the point of interruption, leading to additional processing delays. The *MAD2QN* method achieved the second-best performance with an *Avg. Delay* of 396.54 ms and an *Availability* of 83.43%. However, when it comes to the single-agent approach, the *A2C* method achieved an *Avg. Delay* of 494.52 ms and an *Availability* of 92.47%. This result indicates that decentralized decision-making methods, such as *MAA2C* and *MAD2QN*, are more effective than the single-agent approach in this scenario, as they can better adapt to the system’s dynamic nature and interruptions, and provide better credit assignment for each agent. However, it should be noted that as the number of available choices increases (the number of edge servers increases), the value-based method, such as *MAD2QN*, may suffer from the curse of dimensionality, resulting in suboptimal performance.

For non-DRL methods, when interruptions occur, different strategies handle this situation differently. The *Random* strategy distributes tasks randomly between edge servers and local computation, which can sometimes mitigate excessive loads on specific servers. In contrast, the *LocalEdge* method strictly offloads all device tasks to their respective local edge servers. This approach, although effective under normal conditions, becomes problematic when numerous devices overload the same edge server, resulting in significant computational delays and an overall increase in the average total delay. The *Greedy* method, which prioritizes balancing offloading high-demand tasks to the nearest edge server, performs well since it can distribute the load between edge servers and local devices, and prioritize offloading large tasks with the *Avg. Delay* of 418.23 ms and an *Avail.* of 83.09%, which is approaching the performance of multi-agent DRL-based methods.

Regarding the availability score (*Avail.*), the *OnDevice* method achieved the highest possible availability (100%) because it completely avoided interruptions by performing all tasks locally. However, this resulted in significantly longer computational times. The *MAA2C-based* model had the lowest availability score (83.07%) despite achieving the lowest average total delay (390.95 ms). This is because the agents learn to achieve optimal tradeoffs between reducing delays and maintaining availability. To optimize the overall delay, the model considers the risk of offloading tasks to edge servers, which can sometimes lead to overloads and interruptions, thereby reducing availability.

While they can efficiently learn the balance tradeoff between delay and availability by dynamically adjusting offloading decisions based on the environment, multi-agent DRL-based methods like *MAA2C* and *MAD2QN* suffer from higher standard deviations in average delays compared to more straightforward methods like *Random* and *OnDevice*. While this flexibility can optimize the *Avg. Delay*, it can also cause variability across different episodes or environments, especially when the selected edge servers become overloaded or when interruption events are frequent or unpredictable. This leads to occasional spikes in delays, raising the standard deviation. Sometimes, to achieve lower delay, the *MAA2C-based* method offloads tasks aggressively, increasing the risk of interruption, which can introduce outlier delay values and thereby increase the standard deviation. In contrast, the *OnDevice* method employs a fixed strategy, resulting in a lower standard deviation in average delays.

#### 5.2.2. Simulation Results Using Different Numbers of Edge Servers

**Figure 5:**
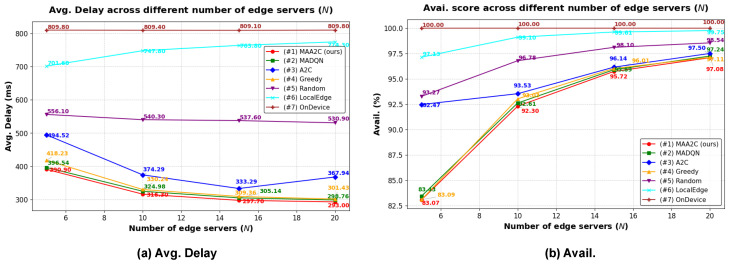
Simulation results using different numbers of edge servers *N*. (**a**) Average total delay. (**b**) Availability score.

In this experiment, we evaluated the performance of the proposed model with different numbers of edge servers *N* while keeping all other hyperparameters set to their default values. The results are presented in Figure 5. As the number of edge servers increased, the average total delay decreased, and the availability score increased. More edge servers enable the devices to offload their tasks to a wider variety of computing node options, thereby reducing the overall load on each server. Consequently, the availability score increased because of the reduced chance of overloading individual servers. The proposed method achieved the lowest Avg.Delay, which reflects an optimal tradeoff for minimizing service delay, although this came at the expense of a lower availability score. In comparison, the *LocalEdge* method achieved the highest availability score (excluding the *OnDevice* method) because a more significant number of edge servers leads to fewer devices per server, which reduces the chance of computational capacity overload. This demonstrates the effectiveness of the proposed method in balancing delay and availability. The *MAA2C*, *MAD2QN*, and *Greedy* methods achieved similar performance with the same trend in terms of Avg.Delay and availability score.

#### 5.2.3. Simulation Results Using Different Numbers of IIoT Devices

**Figure 6:**
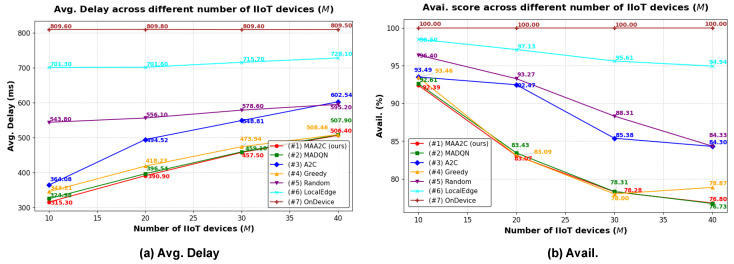
Simulation results using different numbers of IIoT devices *M*. (**a**) Average total delay. (**b**) Availability score.

This experiment evaluated the effectiveness of the proposed model by varying the number of devices *M* while maintaining the other hyperparameters at their default settings. Figure 6 presents the experimental results. For all methods, as the number of devices increased, the average total delay increased, and the availability score decreased. This is because as more devices are used, edge servers are required to handle more tasks, which can lead to computation capacity overload on some servers, consequently increasing the total delay and lowering the availability score. The proposed *MAA2C-based* method achieved the lowest average total delay but also resulted in the lowest availability score, reflecting an optimal tradeoff focused on minimizing the service delay. By contrast, the *LocalEdge* method had the highest availability score after *OnDevice*. This is because a higher number of edge servers distributes the workload more evenly, reducing the risk of overload and improving availability. The *MAA2C*, *MAD2QN*, and *Greedy* methods achieved similar performance with the same trend in terms of Avg.Delay and availability score.

#### 5.2.4. Analysis of the Impact of Edge Server and Device Density on Average Total Delay via Linear Regression

In this experiment, we investigate the relationship between the number of edge servers, *N*, and the number of IIoT devices, *M*, as well as their impact on the average total delay in an interruption-enabled environment. Training the entire MAA2C model for different combinations of (N, M) value pairs can be time-consuming, as the performance of the *Greedy* approach is very similar in trend to MAA2C in terms of average delay (as discussed in Section 5.2.2 and Section 5.2.3). Therefore, in this experiment, we use the Greedy method to collect the average total delay for different (N, M) value pairs, while keeping other parameters at their default settings (as described in Section 5.1.1). The number of edge servers and IIoT devices is modeled using a multiple linear regression approach of the following form:

AverageTotalDelay=aN+bM+c,

where *a*, *b*, and *c* are the coefficients derived from the regression model. This model enables us to analyze the impact of *N* and *M* on the system’s average delay, providing insights into how variations in the number of edge servers and IIoT devices affect overall performance.

To collect the data points for the experiment, we randomly select the number of edge servers (*N*) from 1 to 20 and the number of IIoT devices (*M*) from 1 to 40. Both *N* and *M* are sampled uniformly for 1000 iterations, resulting in 1000 data points. For each pair of *N* and *M*, the *Greedy* method is evaluated for 3000 steps to compute the average total delay.

**Figure 7:**
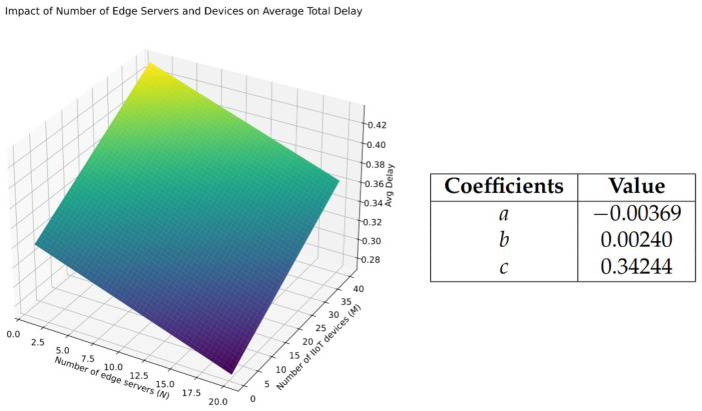
Regression results for average delay based on the number of edge servers (*N*) and IIoT devices (*M*).

Based on the regression results (see Figure 7), we can draw several key insights into how the number of edge servers (*N*) and IIoT devices (*M*) impact the average total delay. The intercept value of 0.3424 represents the baseline delay when both *N* and *M* are zero. Although this scenario is not realistic in practice, it provides a baseline for the model. The coefficient for *N* is −0.0037, which shows that as the number of edge servers increases, the average delay decreases. This is because more edge servers can handle more tasks, as tasks can be distributed, which helps reduce the total load for any edge server, decreases the chance of interruption, and lowers latency. On the other hand, the coefficient for *M* is 0.0024, meaning that as the number of IIoT devices increases, the average delay also increases. This occurs because more devices compete for computational resources, which increases the edge server’s total load and increases the chance of interruptions, resulting in delays. In summary, adding more edge servers helps reduce delays, whereas an increase in IIoT devices tends to worsen delays (as discussed in Section 5.2.2).

#### 5.2.5. Simulation Results Using Different Interruption Sensitivity Values

**Figure 8:**
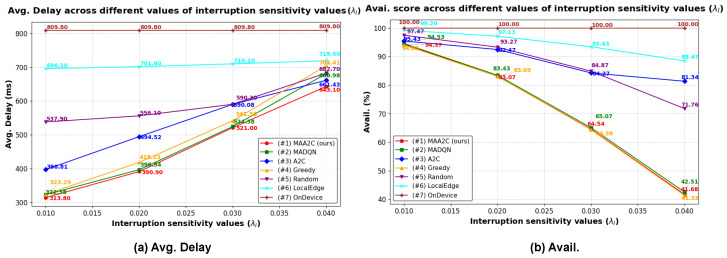
Simulation results using different interruption sensitivity values ($\lambda_{I}$). (**a**) Average total delay. (**b**) Availability score.

This experiment examined the effect of different interruption sensitivity values $\lambda_{I}$ on the performance of the methods. The other hyperparameters were maintained at their default values. The results are shown in Figure 8. As $\lambda_{I}$ increased, the average total delay increased and the availability score decreased owing to a higher load-based interruption probability. This occurred because in our load-based interruption model, $\lambda_{I}$ directly affects the relationship between the total server computation capacity and the probability of interruptions. A higher $\lambda_{I}$ value leads to an increased probability of task interruption, which, in turn, results in higher delays and lower availability. Notably, when $\lambda_{I}$ changed from $2\times 10^{-2}$ to $3\times 10^{-2}$ and $4\times 10^{-2}$, the delay increased significantly and the availability decreased sharply. This rapid increase in *Avg. Delay* occurred because at higher values of $\lambda_{I}$, the system became more sensitive to fluctuations in the server load, causing frequent disruptions in task execution. This increase was even more noticeable when $\lambda_{I}$ reached $3\times 10^{-2}$ or $4\times 10^{-2}$, where the interruption probability increased rapidly, as shown in Figure 2.

#### 5.2.6. Simulation Results Using Different Interruption Durations

**Figure 9:**
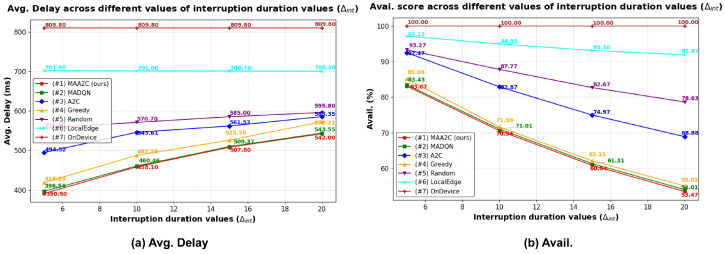
Simulation results using different interruption duration values ($\Delta_{int}$). (**a**) Average total delay. (**b**) Availability score.

In this experiment, we analyzed the impact of different interruption duration parameters $\Delta_{int}$ on system performance across all methods. As seen in Figure 9, when the interruption duration increased, the average total delay increased, and the availability score decreased. This is because the interruption duration represents the time required for an edge server to recover from the interrupted state and return to its normal operation. When an edge server remains interrupted for a long period of time, it becomes temporarily unavailable, forcing the devices to either offload their tasks to other available servers or process them locally.

In our proposed *MAA2C-based* method, a prolonged interruption duration significantly increased the total delay and decreased availability. Because devices must offload their tasks to available edge servers or compute them locally (masking the invalid actions of unavailable edge servers), this can introduce additional processing delays. If too many requests are directed toward the same available edge server, it may become overloaded, further reducing the system availability. This cascading effect can result in severe congestion, leading to a significant decrease in the availability score.

## 6. Conclusions

In this paper, we presented a realistic load-based interruption model in which the probability of server interruption is dynamically defined as an exponential function of the total computational load. This model provides a more accurate simulation of service availability, rendering it suitable for real-world IIoT environments. In addition, we introduced an interruption-aware proactive task offloading framework for IIoT edge computing to address the challenges of task offloading in interruption-prone environments. By leveraging an MAA2C model, our framework guarantees optimal offloading decisions while balancing service latency and system availability. Through extensive experiments, we demonstrated the superiority of our approach over existing methods. Our framework consistently outperformed the baseline strategies in terms of total average delay, especially under dynamic and interruption-sensitive conditions. Furthermore, we analyzed the impact of various system parameters on performance under an interruptible edge task offloading scenario. In the future, we aim to incorporate a dynamic resource allocation strategy into the system to optimize the energy consumption, bandwidth usage, and computational efficiency in IIoT networks. In addition, integrating additional influencing factors beyond the server load, such as network congestion and energy constraints, can further enhance the robustness and real-world reflection of the interruption model.

## Figures and Tables

**Table 1:** Summary of related works. The “✓” symbol denotes the use of an interruption-aware approach.

Paper	Year	Decisions	Approach	Objectives	Interruption Awared	Interruption Model
[13]	2021	- task offloading - delivery	heuristic	delay		
[14]	2022	- task offloading - delivery - resource allocation	heuristic	delay		
[17]	2022	- caching - task offloading - resource allocation	DRL-based	delay		
[15]	2022	- task offloading - resource allocation	heuristic	delay		
[19]	2024	- task offloading	DRL-based	delay		
[24]	2024	- task offloading - resource allocation	game theory	delay		
[20]	2024	- task offloading - relay selection	DRL-based	delay		
[21]	2024	- task offloading	DRL-based	age of information		
[16]	2024	- caching - task offloading - delivery	heuristic	delay		
[23]	2024	- task offloading - resource allocation	MADRL-based	energy		
[22]	2024	- task offloading	MADRL-based	delay		
[28]	2024	- task offloading - resource allocation	MADRL-based	delay, energy		
[12]	2021	- task offloading	greedy	delay	✓	Fixed value
[26]	2022	- task offloading	heuristic	delay, bandwidth	✓	Random-based
[18]	2023	- task offloading	DRL-based	delay	✓	
**Ours**		**- task offloading**	**DRL-based**	**delay**	✓	**Load-based**

**Table 2:** Summary of the parameters used in the simulations.

Parameter	Value	Parameter	Value
**System Parameters**			
Number of IIoT devices *M*	[10,20,30,40]	Edge server total channel bandwidth $B_{E}^{total}$	20 MHz
Number of edge servers *N*	[5,10,15,20]	Edge server sub-channel bandwidth $B_{E}$	2 MHz
Task size $s_{i}\left(t\right)$	[0.5,1] MB	RSU transmission power $P_{R_{j}}$	30
Required CPU cycles $f_{i}\left(t\right)$	[0.5,1] cycles	Noise power $\sigma^{2}$	−174 dBm/Hz
Task deadline $r_{i}\left(t\right)$	[0.8,1] s	Edge-to-edge data rate $R_{E2E}$	150 Mbps
Computation capacity of edge server $F_{E}$	25 GHz	Interruption sensitivity $\lambda_{I}$	[0,0.01,0.02,0.03,0.04]
Maximun computation capacity for each task $F_{E}^{max}$	5 GHz	Interruption duration $\Delta_{int}$	[5,10,15,20] time slots
Computation capacity of IIoT device $F_{D}$	1 GHz	-	-
**Parameters for MAA2C**			
Training step	$10^{5}$ steps	Replay memory size	10,000
Model hidden dimension	128	Batch size	32
Discount factor γ	0.99	Reward scaling factor α, β	100,50
Actor learning rate	$1\times 10^{-5}$	Critic learning rate	$1\times 10^{-2}$

**Table 3:** Simulation results (the best values for each metric are shown in bold).

No	Model	Interruption			
		No ($\lambda_{I}=0$)		Yes ($\lambda_{I}=2\times 10^{-2}$)	
		* Avg. Delay * (ms)	* Avail. * (%)	* Avg. Delay * (ms)	*Avail. *(%)
#1	MAA2C	273.43 ± 20.84	100	**390.95 ± 112.63**	83.07
#2	MAD2QN	323.56 ± 18.18		396.54 ± 110.47	83.43
#3	A2C	297.91 ± 11.79		494.52 ± 81.81	92.47
#4	Greedy	298.75 ± 11.87		418.23 ± 111.84	83.09
#5	Random	392.39 ± 48.55		556.09 ± 66.28	93.27
#6	LocalEdge	**272.70 ± 26.57**		701.59 ± 200.21	97.13
#7	OnDevice	809.84 ± 41.30		809.84± 41.30	100.00

## Data Availability

Data are contained within the article.
